# Saturday Fund Statistics

**Published:** 1892-04-16

**Authors:** 


### Passing Topics.

### SATURDAY FUND STATISTICS.

The report of the Hospital Saturday Fund authorities supplies succinctly and not inopportunely a great deal of information concerning the amount of labour involved in the collection and administration of the Fund. An authenticated statement of this sort is useful beyond the limits of the organisation it is primarily intended to serve, because it brings home to even the casual reader some intimation of the fact, too often forgotten by the critics, that the garnering of funds for hospital or other benevolent purposes is a work of great difficulty calling for an expenditure of both money and energy which is at times altogether disproportionate to the result.

The Saturday Fund report shows that among other workers, it requires a Board, a Council, a Financial Committee, thirty-one district committees, each with its own executive officers, a Treasurer, a Chairman, four honorary Secretaries, a Secretary, and an expenditure of £3,103 to collect and distribute £16,284. While we do not hide our belief that the necessity for all this array, if it be a necessity, is nothing short of discreditable to the large wage-earning class, whose contributions partake of the nature of offerings for benefits directly received, we are quite prepared to acquit the managers of responsibility. Indeed, the amount of effort recorded in the "9,400 visits paid to firms by our organising secretaries," the distribution of 18,272 hospital tickets, and the writing of 20,112 replies to an equal number of letters received, is sufficient in itself to protect those gentlemen from a charge of inactivity, and we fear the soil must be barren, indeed, when all this cultivation ends in so poor a harvest.

If we were disposed to offer adverse criticism in respect to a report which seems to show a large amount of work performed under circumstances of small encouragement, we might say that a total of 222 committee and other meetings held in the course of the year at the offices of the Fund, exclusive, of course, of the district committee meetings, denotes that the larger part of the time of the executive must be expended in a distinctly unprofitable manner. Where can be the use of all this palaver?

If we read between the lines of the report we find indications of an undue prevalence of that familiar desire to have a finger in the pie which is so fertile a source of impotence and failure when individual ambitions are not restrained. Committees composed of large numbers of people with various views, and not a few crotchets, are capable of wasting a great deal of time. Unless very fortunately chosen, the members are apt to concern themselves more with checkmating their colleagues than in co-operating with them, and those who have no particular ideas of their own will often be well content if only they render other people's endeavours sterile.

Notwithstanding the proverbial fate of those who offer advice unasked, we should be disposed to suggest to the Saturday Fund authorities that the very best way of making their organisation the success it ought to be, is to determine upon a plan of selecting the fittest of its following, and of letting the others go. Supervision and control by a body representative enough to ensure confidence are absolutely needful, but no mere mingling of minds has ever produced half the good sense and energy which reside in one active brain, and if the authorities have a capable man at hand, the sooner they learn to trust him the sooner may they look for their fund to illustrate the gratitude rather than the indifference of the working classes. Agricola.

